# Prenatal Diagnosis and Management of a Fetal Goiter Hypothyroidism due to Dyshormonogenesis

**DOI:** 10.1155/2018/9564737

**Published:** 2018-12-19

**Authors:** Catarina Matos Figueiredo, Inês Falcão, Joana Vilaverde, Joana Freitas, Maria João Oliveira, Cristina Godinho, Jorge Dores, Maria Céu Rodrigues, Carmen Carvalho, Teresa Borges

**Affiliations:** ^1^Pediatric Endocrinology Unit, Department of Pediatrics, Centro Materno Infantil do Norte–Centro Hospitalar Universitário do Porto, Oporto, Portugal; ^2^Endocrinology, Diabetes and Metabolism Department, Centro Materno Infantil do Norte–Centro Hospitalar Universitário do Porto, Oporto, Portugal; ^3^Neonatal Intensive Care Unit, Neonatology and Pediatric Intensive Care Department, Centro Materno Infantil do Norte–Centro Hospitalar Universitário do Porto, Oporto, Portugal; ^4^Prenatal Diagnosis Unit, Obstetric Department, Centro Materno Infantil do Norte–Centro Hospitalar Universitário do Porto, Oporto, Portugal

## Abstract

Fetal goiter is a rare disorder not expected to be found during a healthy woman's pregnancy. It can be a prenatal manifestation of congenital hypothyroidism due to thyroid dyshormonogenesis and it can lead to serious perinatal complications. A vascularized fetal neck mass was detected at 29 weeks' gestation of a healthy primigravida. Magnetic resonance was suggestive of goiter causing airway deviation without polyhydramnios. Maternal thyroid function was normal and thyroid antibodies were negative. Two intra-amniotic levothyroxine infusions were performed at 32 and 33 weeks. Serial imaging control showed no progression of the mass. Elective caesarean section was performed at 38 weeks. The male newborn was admitted to the intensive care unit due to cardiorespiratory insufficiency with pulmonary hypertension. Hormonal assays revealed primary congenital hypothyroidism and ultrasonography confirmed diffuse goiter. Levothyroxine was started. Currently, he is 6 years old with adequate growth and normal psychomotor development. Genetic study found a heterozygous mutation in the TPO gene.

## 1. Introduction

Fetal goiter is a very uncommon disorder found during the pregnancy of healthy women without familial thyroid pathology or iodine deficiency. It can be associated with fetal hyperthyroidism and hypothyroidism and even rarely with euthyroid status. Congenital hypothyroidism (CH) is the most frequent congenital endocrine disorder and an usual and preventable cause of intellectual disability [1–3]. The most common cause of CH is thyroid dysgenesis (agenesis, hypoplasia, or ectopy) and only 15-20% is due to specific errors in thyroid hormones synthesis-dyshormonogenesis, which is usually hereditary with autosomal recessive inheritance [2, 4]. Examples of these defects involve mutations in thyroid peroxidase (TPO), thyroglobulin (TG), sodium/iodide transporter, SLC26A4, DUOX2, DUOXA2, and DEHAL1 genes [5, 6].

Fetal goiter can lead to multiple perinatal complications such as polyhydramnios, fetal death, preterm delivery, labor dystocia, neonatal asphyxia, and also long-term morbidity due to neurodevelopmental and growth impairments [5, 7, 8]. The early onset of endocrine substitutive therapy is crucial in the prenatal and also future child outcomes. So far, the main treatment for these situations consists in levothyroxine (L-T4) amniotic infusion; however, there is no agreement regarding in utero therapy in what concerns management and treatment guidelines on which hormones, doses, frequency, and balance between the risks and benefits [2]. There is a reduced number of published cases of hypothyroid fetal goiter and there are no standardized guidelines about this topic [7, 8]. We present a case of fetal goiter as a prenatal manifestation of CH, with airway compression, which underwent L-T4 therapy in utero, emphasizing its rarity, as well as the management difficulties and the severity of the associated potential risks.

## 2. Case Presentation

**Figure 1:**
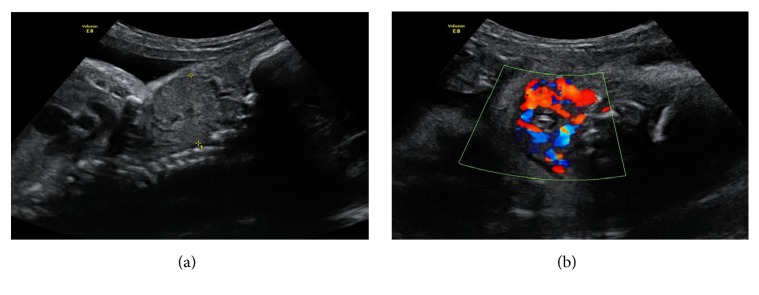
Fetal US by 29 weeks of gestation presenting a vascularized mass in fetal neck.

**Figure 2:**
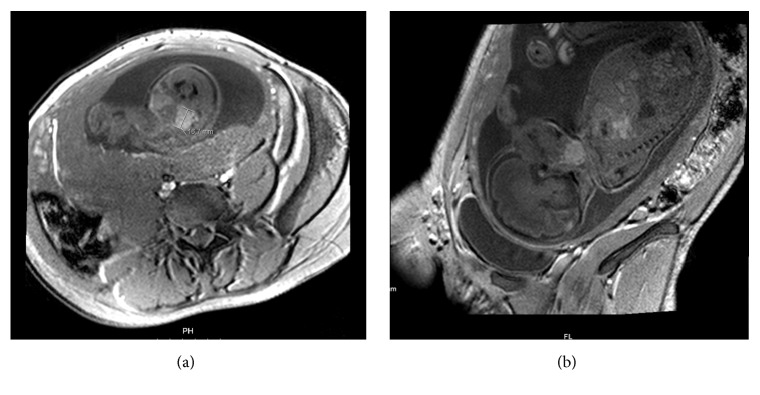
Fetal MRI showing fetal thyroid diffuse enlargement (a: coronal view; b: sagittal view).

A 28-year-old primigravida, without personal thyroid and autoimmune pathology or relevant family history (no consanguinity and unknown endocrine diseases in relatives), underwent prenatal ultrasonography (US) at 29 weeks' gestation, which revealed a high vascularized, bilobed, and symmetric mass in the anterior region of fetal neck (35 mm of largest diameter), suggesting fetal goiter (see Figures 1(a) and 1(b)). No signs of polyhydramnios, cervical hyperextension, and no other fetal anomalies were detected. The mother denied any medication known to interfere with thyroid function and had an adequate diet. Maternal thyroid evaluation showed an euthyroid status without signs of thyroid autoimmunity. To better evaluate the airway patency, a Magnetic Resonance (MRI) was performed at 31 weeks, and it suggested goiter with 39,5x26,7mm, involving and causing airway deviation, with no signs of polyhydramnios (see Figures 2(a) and 2(b)). At 32 weeks, a new US presented a goiter with 35x18x23mm, and first L-T4 amnioinfusion (300 *μ*g-180 *μ*g/kg estimated fetal weight) was performed with concomitant amniotic fluid withdrawal showing increased levels of thyroid-stimulating hormone (TSH) (3,53 *μ*IU/mL, Normal Range (NR): 0,04-0,51 *μ*IU/mL) and normal levels of free thyroxine (fT4) (0.3 ng/dL, NR: 0,10-0,77 ng/dL). A second amniotic L-T4 infusion (400 *μ*g-180 *μ*g/kg estimated fetal weight) was performed ten days later; at that time goiter showed 36x24x24mm and amniotic hormonal levels were TSH 1,69 *μ*UI/ml (NR: 0,04-0,51 *μ*UI/mL) and fT4 0.6 ng/dL (NR: 0,10-0,77 ng/dL). Serial imaging control did not show goiter size reduction, including last US at 37 weeks with 35x32x27mm, but also did not reveal the development of complications such as polyhydramnios.

Elective cesarean section was performed at 38 weeks of gestational age, and a male neonate was delivered with Apgar scores of 7/9 at first and fifth minutes, weighting 3480 g, showing a palpable goiter and exhibiting some breathing difficulties. He was promptly admitted to the neonatal intensive care unit due to respiratory distress and increasing oxygen requirements with cardiorespiratory insufficiency, moderate pulmonary hypertension, and decreased ventricular function requiring mechanical ventilation and aminergic support. Hormone assays of umbilical cord blood confirmed primary CH with reduced fT4 (0.2 ng/dL NR: 2,00-5,00 ng/dL), elevated TSH (715 *μ*IU/mL NR: 2,3-13,2 *μ*IU/mL), TG (4376 ng/mL NR: 14,7-101,1 ng/mL), and absence of thyroid autoantibodies. Thyroid replacement therapy with L-T4 was promptly started in the first hours of life, at a dose of 10 *μ*g/kg/day. Biochemical control at fourth day of postpartum showed an increasing of fT4 to 0,9 ng/dL and a reduction of TSH to 103,8 *μ*IU/mL.

Postnatal cervical US revealed an enlarged, slightly hypoechoic, and heterogeneous thyroid gland (right lobe: 18x32x18mm; left lobe 18x38x17mm) corroborating prenatal goiter diagnosis. Mechanical ventilation was maintained until the fifth day of life, and aminergic support was discontinued by the sixth day. Clinical evolution was favorable with discharge home at D12 with outpatient pediatric endocrinology follow-up.

He failed the newborn hearing screening by otoacoustic emissions; however hearing loss was not confirmed in the evoked auditory potentials. Genetic study found two pathogenic variants, both heterozygous, in TPO gene [c.1472G>A(;)1993C>T].

Currently, he is six years old with adequate growth without cognitive deficits (the Development Quotient score according to the revised Griffiths' scale was 100 at 44 months, which corresponds to the average level expected for age). He presents goiter with heterogeneous structure without focal lesions and is still under L-T4 treatment, adjusted according to serial hormonal monitoring.

## 3. Discussion

Prenatal diagnosis of fetal goiter, its correct investigation, and management are challenging. Multiple causes must be considered in fetal neck masses investigation, such as cystic hygroma, teratoma, angioma, lymphangioma, and goiter, among others. Thus, imaging exams like US and, when not well clarified, fetal MRI assume a relevant role in clarifying the underlying cause [1, 6, 7]. After the fetal goiter diagnosis, initial assessment should include maternal medication or supplementation, iodine status, thyroid function, and autoimmune thyroid disorders (TSH, fT3, fT4, anti-TPO, anti-TG, and TSH receptor blocking antibodies) [1, 7].

The most frequent cause subjacent to the hypothyroid fetal goiter is the maternal thyroid dysfunction and its medications, being very rare in situations of euthyroid mothers. Another possible reason is maternal intake of iodine supplements or endemic iodine deficiency [7, 8]. Our case represents an example of hypothyroid fetal goiter in an euthyroid mother. Given the fact that maternal information was not suggestive, maternal iodine measuring was not performed.

As fetal goiters are associated with fetal hyperthyroidism and hypothyroidism and rarely with euthyroidism, fetal thyroid function assessment is recommended. Cordocentesis remains the gold standard and it is the preferred and more accurate method, although it is technically more difficult to perform and it carries further pregnancy risks such as cord bleeding, bradycardia, intrauterine infection, preterm labor, and fetal death. Amniocentesis is better accepted by parents when both methods are proposed to assess fetal thyroid status. However, experts have expressed some doubts about accuracy and correlation between thyroid hormonal levels in amniotic fluid and fetal hormonal status [1, 6, 7].

Another concern refers to the management of possible complications associated with fetal cervical mass itself. These include esophageal compression which can lead to polyhydramnios, neck hyperextension leading to malpresentation, and difficult delivery with the risk of labor dystocia and newborn asphyxia. Finally, it may cause newborn airway compression with possible respiratory distress and more complicated intubation and ventilation.* Vasudevan et al*. described a fatal intrauterine outcome of a hypothyroid fetal goiter due to severe polyhydramnios, even after L-T4 therapy, without evidence of infection [5].* Mastrolia *et al. presented a newborn with tracheal involvement at birth and need for mechanical ventilation despite prenatal therapy [7]. Our case is an example of neonatal respiratory distress with significant acute morbidity as there was a need of ventilatory and aminergic support. The relationship between pulmonary hypertension and hyperthyroidism has been well described in the literature. However, it has not been well established with hypothyroidism, except for NKX2-1-related disorder. This is also known as brain-lung-thyroid syndrome and manifests with childhood-onset chorea, CH, and neonatal respiratory distress [9]. The initial respiratory distress showed in the immediately neonatal period of our case was interpreted as resulting from a complication of goiter itself linked to upper airway obstruction. However, other factors could influence the hemodynamic status such as the role of the thyroid hormones in lung epithelial cells differentiation, lung maturation and alveolar septation, or the low-resistance arteriovenous shunt in the systemic circulation due to goiter itself [10].

It should be noticed that there is no consensus regarding who should be treated or when the treatment should be started, which hormone to use, appropriate dose, number of administrations, and the interval between them [1, 7]. Treatment is controversial because this pathology is rare and there are few published cases reviews.

Assuming a limited transplacental passage of fT4 and the fact that the fetus swallows the amniotic fluid, it is considered that by increasing intra-amniotic L-T4 levels, increased fetal L-T4 levels and reduced goiter size can be achieved. Treatment with intra-amniotic injections of L-T4 has been preferable than L-T3. However, difficulties in the acquisition and authorization for the use of parenteral L-T4 have been mentioned, sometimes causing a delay in the beginning of therapy or the use of L-T3 until the situation was solved [3, 5]. Some authors suggest in utero therapy only for situations of fetal goiter with progression or complications development such as polyhydramnios. Thus, slow-growing or stable goiters can be managed conservatively, with serial imaging follow-up, avoiding invasive intrauterine and repetitive procedures, due to inherent risks [6, 11].

The primary purpose of prenatal treatment consists in the reduction of goiter size to enable pregnancy to come to term without perinatal complications [7]. The literature has demonstrated efficacy in reducing goiter size [1, 2]; however, it also showed some adverse consequences, such as preterm labor and chorioamnionitis [4, 5, 7]. In our case, we did not achieve an absolute fetal goiter size reduction, although the relative proportion of goiter significantly reduced as fetal growth occurred; nevertheless, stabilization of thyroid growth trend was accomplished, so we were able to prevent prenatal complications and enable a term delivery. The decision to treat must take into account the benefit-to-risk analysis of these repeated procedures, which have significant fetal morbidity.

As in most published cases, our case had a severe hypothyroidism at birth in spite of therapy instituted in utero.* Stewart *et al. described a rare case of CH with euthyroid status at birth after prenatal treatment [3]. The timing of the latest injection before birth has been mentioned as an important determinant of newborn thyroid status [1, 7, 8]. In our case, this could justify the treatment failure to achieve euthyroidism at birth due to the long period between the last injection and birth (4 weeks). Prompt hormonal replacement therapy after birth is crucial to optimize prognosis. This case also shows the ability to grow properly without neurodevelopment cognitive impairment with accurately adjusted therapy.

In the presence of fetal goiter in a euthyroid mother and CH, we suspected of dyshormonogenesis, which was confirmed by genetic studies that revealed two heterozygous and pathogenic variants in the TPO gene. These situations justify genetic counseling since it helps to predict risk of recurrence and management of prenatal treatment [1].

## 4. Conclusions

We present a case of CH due to thyroid dyshormonogenesis manifested by fetal goiter and emphasize the management difficulties on account of the lack of consensual guidelines. Prenatal diagnosis of fetal cervical mass requires a careful and permanent investigation, as it can imply important decisions and therapy even during intrauterine life. Early diagnosis and prompt therapy are essential to optimize prognosis.
